# DKK1 promotes hepatocellular carcinoma cell migration and invasion through β-catenin/MMP7 signaling pathway

**DOI:** 10.1186/1476-4598-12-157

**Published:** 2013-12-10

**Authors:** Liang Chen, Ming Li, Qian Li, Chao-jie Wang, Song-qiang Xie

**Affiliations:** 1 Institute of Chemical Biology, Pharmaceutical College of Henan University, Kaifeng 475004, China; 2 The Key Laboratory of Natural Medicine and Immuno-Engineering, Henan University, Kaifeng 475004, China

**Keywords:** DKK1, Hepatocellular carcinoma, Migration, Invasion, β-catenin, MMP7

## Abstract

**Background:**

Recently several reports have indicated that elevated expression of DKK1 is tightly associated with the progression of hepatocellular carcinoma (HCC). However, the biological function of DKK1 in HCC has not yet been well documented.

**Methods:**

In this study, the role of DKK1 in tumor cell proliferation, migration and invasion was investigated using MTT, colony formation, wound scratch, transwell assays, and also human HCC samples.

**Results:**

Both gain- and loss-of-function studies showed that DKK1 did not influence the tumor cell proliferation and colony formation, while dramatically promoted HCC cell migration and invasion. Subsequent investigations revealed that β-catenin was an important target of DKK1. The blocking of β-catenin by pharmacological inhibitor antagonized the function of DKK1, whereas introduction of β-catenin by transfection with plasmids or treatment with GSK3β inhibitor phenocopied the pro-migration and pro-invasion effects of DKK1. We further disclosed that DKK1 exerted its pro-invasion function, at least in part, by promoting β-catenin expression, in turn, upregulating the expression of matrix metalloproteinase 7 (MMP7), which was independent of the canonical Wnt signaling pathway. Moreover, introduction of MMP7 significantly enhanced the ability of HCC cells to invade extracellular matrix gel *in vitro*. Consistently, in human HCC tissues, DKK1 level was positively correlated with β-catenin expression, as well as tumor metastasis.

**Conclusion:**

Taken together, these results demonstrated that DKK1 is overexpressed in HCC; moreover, ectopic expression DKK1 promotes HCC cell migration and invasion at least partly through β-catenin/MMP7 signaling axis, suggesting that DKK1 may be a promising target for HCC therapy.

## Background

Hepatocellular carcinoma (HCC) is one of the most commonly diagnosed cancers, ranking as the third leading cause of all cancer-related death in the world [1]. Despite some advances in early detection and the use of modern surgical techniques in combination with various treatment modalities, such as radiotherapy and chemotherapy, to date, the prognosis of the patients with HCC still remains poor. It has been shown that only 30%-40% of patients are amenable to potentially curative therapies, because most patients are often at advanced stage of disease at the time of diagnosis [2]. Moreover, the overall 5-year survival rate of HCC patients remains unsatisfactory, mainly because of a high incidence of recurrence and metastasis after hepatic resection [3]. It is well-believed that elucidation of the underlying molecular basis involved in HCC progression and metastasis is very important for the development of effective HCC therapeutic agents. In order to improve HCC treatments and outcomes, therefore, identifying the potential molecules that promote HCC progression and metastasis are urgently needed.

Dickkopf1 (DKK1), a secreted protein involved in head formation in embryonic development, binds to the low-density lipoprotein receptor-related protein-5/6 (LRP5/6) Wnt co-receptor and prevents the formation of active Wnt-Frizzled-LRP5/6 receptor complexes, thus blocking the canonical Wnt/β-catenin signaling pathway [4]. Numerous studies have shown that DKK1 was implicated in the control of various pathological and physiological processes, including adult hippocampal neurogenesis [5], osteoclastogenesis [6], tumor cell proliferation, survival, migration and invasion [7,8]. However, the role of DKK1 in tumor biology is controversial. Several studies have shown that DKK1 can either inhibit or promote tumor progression and metastasis. On one hand, DKK1 has been shown to inhibited tumorigenesis. For example, González-Sancho *et al.* found that DKK1 expression decreases in human colon tumors, suggesting that DKK1 may act as a tumor suppressor gene in this neoplasia [9]. Indeed, up-regulation of DKK1 causes a decrease in colon cancer cell proliferation, clonogenicity, migration, and invasiveness [10,11]. On the other hand, overexpression of DKK1 was found in 126 out of 180 human non-small cell lung cancers, 59 of 85 small cell lung cancers, and 51 of 81 esophageal squamous cell carcinomas patients [12]. High expression of DKK1 has also been reported in breast and kidney cancers [13]. A recent research article showed that high expression of DKK1 is related to lymphatic metastasis and indicates poor prognosis in intrahepatic cholangiocarcinoma (ICC) patients after surgery, vice versa, depletion of DKK1 using small interfering RNA results in a decrease in ICC cell migration and invasion [8]. Taken together, all these findings suggest that DKK1 performs an oncogenic or a tumor-suppressing function depends on the cell type or context.

Several years ago, Qin *et al.* reported that overexpression of DKK1 by transfection is able to inhibit the growth and migration. But vice versa, reduction of DKK1 expression by RNA interference is able to increase the migration in a model of hepatocellular carcinoma cells [14]. However, recently several research studies indicated that elevated expression of DKK1 was found in both tissue and serum samples from patients with HCC [15-17]. Moreover, overexpression of DKK1 not only enhances the tumor formation efficiency and tumor growth but also promotes the cell invasion and metastasis *in vitro* and *in vivo*, whereas knockdown of DKK1 significantly reduced both migratory and invasive abilities of HCC cells [15,17]. Therefore, whether DKK1 is an oncogene or a tumor suppressor in HCC remains to be further investigated. Furthermore, the precise mechanism of DKK1 involved in tumorigenesis has not been well-documented.

In this present study, both gain- and loss-of-function studies showed that DKK1 did not influence HCC cell proliferation and colony formation, while dramatically promoted HCC cell migration and invasion. We further confirmed that DKK1 exerted its pro-invasion function, at least in part, by promoting β-catenin expression, in turn, upregulating the expression of matrix metalloproteinase 7 (MMP7), which was independent of the canonical Wnt signaling pathway. Moreover, introduction of MMP7 significantly enhanced the ability of HCC cells to invade extracellular matrix gel *in vitro*. Our findings highlight the oncogenic role of DKK1 in promoting HCC cell migration and invasion through activation of β-catenin/MMP7 signaling pathway, implicating that DKK1 may serve as a potential target for HCC therapy.

## Materials and methods

### Materials

3-(4, 5-dimethylthiazol)-2, 5-diphenyltetrazolium bromide (MTT), Hoechst33342, G418 and LiCl were purchased from Sigma (St. Louis, MO, USA). RPMI 1640 and fetal calf serum (FCS) were purchased from Gibco (Grand Island, NY, USA). The sources of primary antibodies used for Western blot: DKK1 (cat. sc-25516), LRP6 (cat. sc-17982), GSK3β (cat. sc-53931), β-catenin (cat. sc-7199) and MMP7 (cat. sc-101566) as well as the corresponding horseradish peroxidase-conjugated second antibodies were all purchased from Santa Cruz Biotechnologies (Santa Cruz, CA, USA). Cy3-labeled goat anti-mouse second antibody was purchased from Beyotime Institute of Biotechnology (cat. A0521, Shanghai, China). All other chemicals used in this study were commercial products of reagent grade.

### Human tissue specimens and cell lines

Human HCC (30 cases of primary, 41 cases of metastasis) and adjacent non-tumor liver tissues were collected from 71 patients undergoing resection of HCC at the Huai-He Hospital, Henan University (Kaifeng, China). All tissues were classified according to the tumor-node-metastasis classification system of the union for International Cancer Control. Informed consent was obtained from each patient, and the study was approved by the Institute Research Ethics Committee at the hospital.

Cell lines, derivative from human HCC (HepG2, SMMC7721, Huh7 and Bel7402) and normal liver cell line (QSG-7701) were used in this study. All of them were purchased from the cell bank of the Chinese Academy of Science (Shanghai, China), these cells were maintained in RPMI1640 medium containing 10% fetal calf serum, 100 units/mL penicillin, and 100 μg/mL streptomycin, at 37°C in a humidified incubator containing 5% CO_2_.

### Plasmid construction

The full-length coding region (801 bp) of DKK1 was amplified from human genomic DNA by reverse transcription-polymerase chain reaction (RT-PCR) using the following primers: DKK1-sense 5′-CCG CTC GAG ATG ATG GCT CTG GGC GCA GCGGG-3′, and antisense 5′-CGG GAT CCG CTG GTT TAG TGT CTC TGA CAAGT-3′, then the PCR products were digested with XhoI/BamHI and were inserted into the pIRES2-EGFP vector (Clontech).The recombinant construct was verified by direct DNA sequencing.

For the construction of siRNA expression plasmids, A siRNA sequence targeted human DKK1 transcript (accession no. NM_012242.2; sense 5′-GGAATAAGTACC AGACCATTG-3′) was selected for RNA interference (RNAi). This sequence showed no homology with other known human genes. A scrambled sequence (sense 5′-GGAATAAGACCATGACCATTG-3′), which is no homologous to any human DNA sequence, served as a negative control. Two complementary oligonucleotides that contain both sense and antisense siRNA sequences, the loop sequence (5′-TTCAAGAGA-3′) and the flanking B*am*H I and E*co*R I sites were synthesized chemically. Then these two complementary oligonucleotides were annealed and ligated into the linearized pSIREN-Shuttle (Clontech). Each recombinant construct was sequenced to confirm the right sequence of insert. The construct contained siDKK1 sequence was designated as pSIREN-Shuttle-siDKK1, while the plasmid with scrambled sequence was named as pSIREN-Shuttle-Control.

### Transfection

For transfection, HepG2 and Bel7402 cells were seeded in 24-well plates at 5 × 10^4^ per well and incubated at 37°C in a humidified incubator containing 5% CO_2_. The next day, when these cells were about 80% confluence, cells were transfected with TurboFect™ *in vitro* Transfection Reagent according to the manufacturer’s instructions. These transfected cells were selected with 0.1 mg/mL G418 for at least 2 weeks, and then the stable plasmid-transfected clones were generated by using limiting dilution analysis in 96-well plates. The clones derived from HepG2 cells stably transfected with pIRES2-EGFP-DKK1 or pIRES2-EGFP vector were classified as DKK1 and Vector respectively; whereas the clones derived from Bel7402 cells stably transfected with pSIREN-Shuttle-siDKK1 or pSIREN-Shuttle-Control were classified as shDKK1 and shControl respectively. For β-catenin and MMP7 transfections, tumor cells transiently transfected with human beta-catenin pcDNA3 (plasmid 16828; Addgene, Cambridge, MA), pcDNA3 (Invitrogen), pCMV6-XL5-MMP7 and pCMV6-XL5 (OriGene, USA) using TurboFect™ *in vitro* Transfection Reagent for 36 h, then were applied to other expriements.

### Cell growth curve analysis

The MTT assay was used to detect the proliferation rate of tumor cells. Briefly, 2000 cells per well were plated in 96-well plates and incubated 1, 2, 3, 4, 5, 6 and 7d, respectively. At indicated time point, the process was performed as described before [18]. Briefly, 50 μl of MTT reagent (1 mg/mL) was added and incubated for 4 h at 37°C in a humidified incubator containing 5% CO_2_. Supernatants were removed from the wells, and then 100 μl DMSO was added to solubilize the crystal products at room temperature for 10 min. The absorbance (OD) was measured with a microplate reader (Bio-Rad) at a wavelength of 570 nm.

### RNA isolation and RT-PCR

For PCR, total RNA was extracted from sub-confluent using TRIzol reagent (Invitrogen). Two microgram of total RNA was subjected to DNase I digestion (1 U/μL, Fermentas, Hanover, MD) at 37°C for 30 min, and then the DNase I was heated inactivation at 70°C for 15 min, followed by reverse-transcription using PrimeScript™ Reverse Transcriptase (Takara). Semiquantitative RT-PCR was performed using primers listed in Additional file 1: Table S1. All PCR reactions were done using the following conditions: 95°C 5 min, 95°C 45 s, annealing at different temperatures for each gene respectively 45 s, extension 72°C 1 min for 30 cycles, and a final extension at 72°C for 10 min. All PCR products were separated by electrophoresis on 1.0% agarose gels.

### Colony formation assay

The colony formation assay was performed as previously described with some modification [19]. Briefly, a total of 400 cells every well were seeded into a fresh 6-well plate and incubated in RPMI1640 containing 10% FCS, cell medium was changed every 3 d for 15 d until visible colonies formed. Colonies were fixed with methanol and stained with 0.1% crystal violet in 20% methanol for 20 min. Microscopic colonies composed of more than 50 cells were counted in each well.

### TCF luciferase reporter assay

HCC cells grown in 24-well plates were transiently transfected with M50 Super 8x TOPFlash with the wild-type TCF binding sites or its control M51 Super 8X FOPflash with the mutant TCF binding sites ((plasmid 12456 or plasmid 12457, Addgene, Cambridge, MA), in combination with pRL-TK (Renilla luciferase; Promega, Madison, WI) using TurboFect™ *in vitro* Transfection Reagent (Fermentas) according to the manufacturer’s instructions. After 36 h, the transfected cells were lysed and luciferase activity measurement was performed with the Dual-Luciferase Reporter Assay System (Promega).

### Wound scratch assay

The wound scratch assay was performed as described previously [15]. Briefly, cells were plated in a 24-well plate and grown to a confluence cell monolayer overnight prior to serum starvation for 24 h. After scratching with a pipette tip (20 μL); in order to eliminate the floating cells, culture medium was removed and wells were washed three times with PBS. The width of the wound was measured and recorded as t = 0. The cells were then allowed to migrated back into the wounded area, 24 h latter, the width of the open area was measured. Cell migration was expressed as the percentage of the gap (t = 24 h) relative to the primary width of the open area (t = 0 h). All experiments were performed in triplicate.

### Cell migration and invasion assay

The migration and invasion assays were performed as in a 24-well Boyden chamber with 8 μm pore size polycarbonate membrane (Corning, NY, USA), as previously described [20]. For migration assay, 200 μL of serum-free medium (containing 1 × 10^5^ cells) was added to the upper compartment of the chamber, while the lower compartment was filled with 600 μL of RPMI 1640 supplemented with 10% FBS. After incubation at 37°C for 24 h, the tumor cells remaining inside the upper chamber were removed with cotton swabs. The cells on the lower surface of the membrane were stained with 0.1% crystal violet after fixation with methanol, and then counted under a light microscope. The invasion assay was done by the same procedure, except that the membrane was coated with Matrigel to form a matrix barrier, and 3 × 10^4^ tumor cells were added to the upper chamber.

### Immunofluorescence microscopy

The immunofluorescence staining was carried out as previously described with some changes [20]. Briefly, tumor cells were washed three times with ice-cold PBS, fixed in 4.0% paraformaldehyde. After permeabilization with 0.1% Triton X-100, cells were blocked of nonspecific binding by incubation with 5% normal goat serum in PBS. Afterwards, cells were incubated with the rabbit polyclonal β-catenin antibody overnight at 4°C. The staining was completed with Cy3-labeled goat anti-rabbit second antibody (1:500, Beyotime, China) incubation for 1 h at room temperature, and nuclei was counterstained with Hoechst33342 (10 μg/mL, Sigma, USA) for 30 min at dark. All photographs were taken by using High content screening (HCS) (Thermo Scientific Cellomics ArrayScan Vti, Cellomics, Inc., Pittsburgh, PA).

### Western blot analysis

Western blot analysis was performed as our described previously [18]. Briefly, cell pellets were washed thrice with ice-cold phosphate buffered saline (PBS) and were lysed with RIPA buffer (Beyotime, China). The total protein concentration was determined using a BCA assay kit (Beyotime, China). Samples were denatured in 5 × SDS-sample buffer at 95°C for 5 min. Equal amounts of total proteins were separated using 12% SDS-PAGE, and then transferred onto PVDF membranes. Membranes were then blocked by 5% dried skimmed milk in PBST at room temperature for 1 h. After blocking, membranes were incubated with corresponding primary antibodies overnight at 4 °C. After washed three times with PBST, the membranes were incubated with appropriate HRP-conjugated secondary antibody, and then washed three times with PBST. Proteins were detected by using the ECL plus reagents (Beyotime, China).

### Immunohistochemical (IHC) staining

Formalin-fixed and Paraffin-embedded tissue sections were de-paraffinized in xylene, rehydrated through graded ethanol, quenched for endogenous peroxidase activity in 0.3% hydrogen peroxide, and processed for antigen retrieval in citrate buffer (pH 6.0) for 10 min by microwave heating. Following overnight incubation at 4°C using rabbit mAb against human DKK1 (1:200) or β-catenin (1:100). Immunostaining was performed with an optimal amount of HRP-conjugated secondary antibody for 1 h at room temperature and DAB was used as chromogen. Subsequently, sections were counterstained with hematoxylin.

DKK1 and β-catenin expression were evaluated under a light microscope at a magnification of 200×. For each specimen, five images of representative areas were acquired and tumor cells were counted. For human samples, IHC scoring was performed using a modified Histo-score (H-score) as described by Fang *et al.*[21].

### Statistical analysis

Data in this study were expressed as mean ± SEM from at least three separate experiments. Unless otherwise noted, the differences between groups were analyzed using Student’s t-test (two groups) or ANOVA (≥ three groups). All statistical analyses were performed using SPSS 13.0 software (Chicago, IL, USA). All tests employed were two-sided, and p <0.05 was set to be considered statistically significant.

## Results

### DKK1 is overexpressed in human hepatocellular carcinoma cell lines

**Figure 1:**
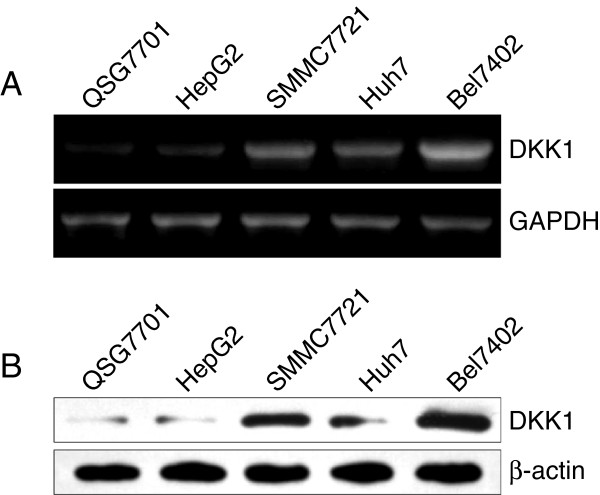
**DKK1 is overexpressed in HCC cells. A**: The mRNA expression of DKK1 in normal liver cell line (QSG7701) and HCC cell lines (HepG2, SMMC7721, Huh7 and Bel7402). GAPDH was used as an internal control. **B**: Western blot analysis of DKK1 in the same cell lines as described in A. β-actin served as a loading control.

To determine the possible role of DKK1 in human HCC, the levels of DKK1 mRNA in four different hepatocellular carcinoma cell lines were compared with that in the normal liver cell line by using RT-PCR. We observed that the mRNA expression of DKK1 in Huh7, SMMC7721, and Bel7402 cell lines were much higher in comparison with that in control normal QSG7701 hepatocytes (Figure 1A). We further investigated the protein expression of DKK1 in these cells by western blotting. As shown in Figure 1B, the higher protein level of DKK1 was confirmed in Huh7, SMMC7721, and Bel7402 HCC cell lines, compared with those of QSG7701 cells. Of note, Bel7402 hepatocellular carcinoma cells exhibited the highest level of DKK1 in both mRNA and protein level. In contrast, the level of DKK1 mRNA and protein in HepG2 cells was comparable to that in the normal liver cell line (QSG-7701). Therefore, HepG2 and Bel7402 cells were selected for further study.

### DKK1 overexpression did not influence the cell proliferation rate and colony formation

**Figure 2:**
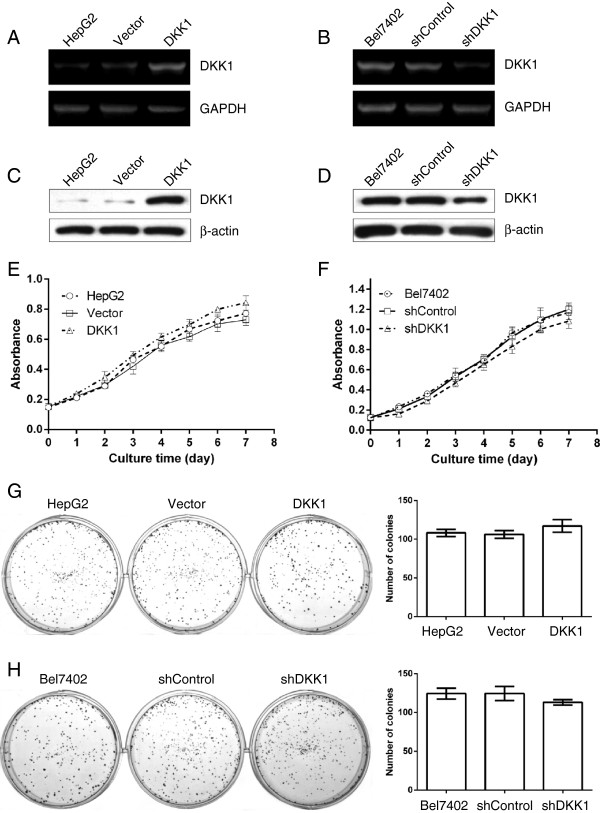
**DKK1 up-regulation or down-regulation did not influence HCC cell proliferation.** The expression of DKK1 mRNA **(A, B)** and protein **(C, D)** were detected in HepG2 cells without transfection (lane 1) or transfected with Vector (lane 2) or DKK1 (lane 3), or Bel7402 cells without transfection (lane 1) or tumor cells transfected with shControl (lane 2) or shDKK1 (lane 3) using RT-PCR and western blotting. GAPDH and β-actin were used as loading controls. **E, F**: MTT assay showed that up-regulation or down-regulation of DKK1 did not influence the proliferation rates in HepG2 or Bel-7402 cells. **G, H**: enforced expression or suppression of DKK1 did not influence the colony formation in both HepG2 and Bel7402 HCC cells. HepG2 cells without transfection or transfected with Vector or DKK1 **(G)**, and Bel7402 cells that without transfection or transfected with shControl or shDKK1 **(H)** were seed into 6-well plates and incubated in RPMI1640 supplemented with 10% FCS, the medium was changed every three days. 15 days later, colonies were fixed and stained with 0.1% crystal violet in 20% methanol for 20 min. Colonies composed of more than 50 cells were counted in each well. The data are represented as means ± SEM from 4 replicates in each of the 3 independent experiments.

Next, we asked for the functional role of DKK1 in tumor cells. After stably transfected cells were individually selected, the DKK1 and the housekeeping gene GAPDH mRNA levels were measured using RT-PCR. Compared with the cells which without transfection or transfected with Vector, the levels of DKK1 mRNA were greatly up-regulated in HepG2 cells transfected with DKK1 (Figure 2A). However, the tumor cells derived from shDKK1-tranfectants showed a much lower level of DKK1 mRNA in comparison with that derived from shControl or nontransfected Bel7402 cells (Figure 2B). Consistent with our RT-PCR data, Western blot analysis also revealed that DKK1 protein levels were also obviously increased in HepG2 cells that transfected with DKK1 (Figure 2C), while decreased in Bel7402 cells that transfected with shDKK1, compared with their control groups respectively (Figure 2D). The empty vector or the control scrambled shRNA did not substantially affect the endogenous DKK1 expression in both mRNA and protein levels. Collectively, these findings indicated that DKK1 can be stably overexpressed in HepG2 cells and shDKK1 could effectively suppress the expression of DKK1 in Bel7402 cells. Then we investigated whether DKK1 regulates cell proliferation. When DKK1 was up-regulated in HepG2 cells, no change of proliferation rates occurred compared with that without transfection or transfected with Vector (Figure 2E). Next, we aimed to investigate if loss of DKK1 may influence tumor cell proliferation. Therefore, DKK1 was depleted using one DKK1-specific shRNA and proliferation was assessed in Bel7402 cells, which expressed high levels of DKK1 endogenously. In these cells, remain no obvious change of proliferation rates were observed (Figure 2F). Using colony formation assay, although DKK1-transfectants displayed a tendency of increased colonies (Figure 2G), and shDKK1-transfectants showed a reduced tendency (Figure 2H), however, the difference did not reach statistical significance, compared with their control cells respectively.

### DKK1 promotes HCC cell migration and invasion

**Figure 3:**
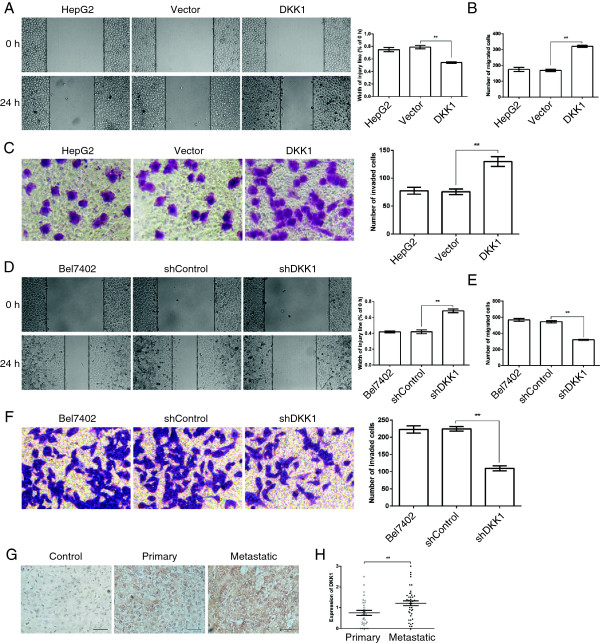
**DKK1 was involved in HCC cell migration and invasion. A**: Wound healing assay indicated that DKK1 up-regulation promoted HepG2 cell migration. **B, C**: Up-regulation of DKK1 significantly enhanced HepG2 cell migration and invasion. HepG2 cells without transfection or transfected with Vector or DKK1 were added to the transwell chamber coated without or with Matrigel for 24 h. Representative images and the number of migrated or invaded cells are shown. Analysis was performed in three separate experiments (n = 3). **, p <0.01. **D**: Wound healing analysis of migration in Bel7402 cells. **E, F**: Depleting of DKK1 attenuated the migration and invasion in Bel7402 cells. Bel7402 cells without transfection or transfected with shControl or shDKK1 cells were seeded into the transwell chamber coated without or with Matrigel and incubated in RPMI1640 containing 10% FCS for 24 h. and then fixed and stained with crystal violet for 20 min. Representative images and the number of migrated or invaded cells are shown. Results are presented as mean ± SEM (n = 3). **, p <0.01. **G, H**: Analysis of DKK1 expression in primary (n = 30) and metastatic (n = 41) HCC tissues by IHC. Images were captured at × 200 for DKK1 staining. Scale bar = 50 μm. The data are represented as means ± SEM. **, p <0.01.

Given that DKK1 did not influence tumor cell proliferation or colony formation, then we investigated whether DKK1 regulated HCC cell migration and invasion, which are two of the most important features of malignant cell behavior. First, we examined the role of DKK1 in tumor cell migration by using wound scratch assay. As illustrated in Figure 3A, enforced expression of DKK1 significantly promoted wound closure compared with that without transfection or transfected with Vector in HepG2 cells, vice versa, down-regulation of DKK1 remarkably attenuated the wound closure (Figure 3D). To further identify these observations, we examined the effects of DKK1 on HCC cell migration using the Boyden chamber transwell without Martrigel. Indeed, tumor cells derived from DKK1-transfectants displayed a higher ability of migration, compared with that derived from Vector or nontransfected cells (Figure 3B). In contrast, tumor cells derived from shDKK1-transfectants displayed a lower ability of migration (Figure 3E). Next, we determine the role of DKK1 in tumor cell invasion. We performed these experiments using transwell chamber with Matrigel. As illustrated in Figure 3C, up-regulation of DKK1 expression significantly enhanced the number of HepG2 cells that invaded through Matrigel in comparison with that derived from Vector or nontransfected cells, however, the number of Bel7402 cells that invaded through Matrigel was sharply reduced after DKK1 was silenced by a specific shRNA (Figure 3F). Consistently, in the human HCC tissues, the primary tumors showed slightly higher DKK1 expression, compared to those in the adjacent non-tumor liver tissues, moreover, the metastatic tumors showed much higher DKK1 expression in comparison with that in the primary tumors (Figure 3G and H). Collectively, these data demonstrate that DKK1 promotes HCC cell migration and invasion *in vitro* and tumor metastasis *in vivo*.

### DKK1 regulates the expression of β-catenin

**Figure 4:**
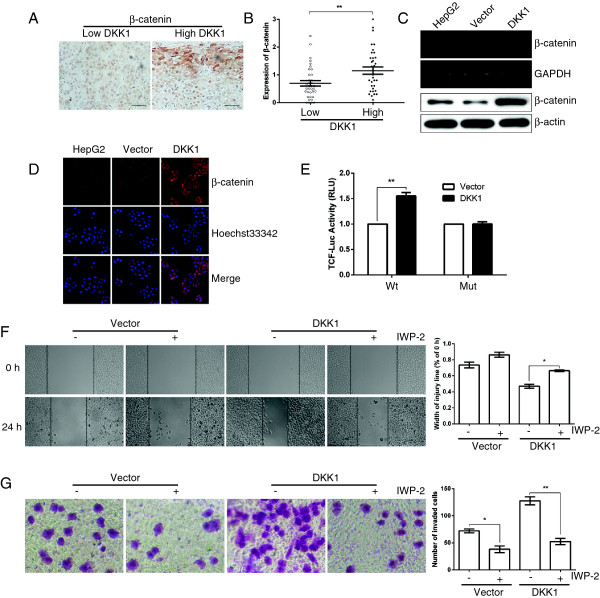
**Upregulation of DKK1 promotes the expression of β-catenin. A**: The representative images of IHC staining for β-catenin. Scale bar = 50 μm. **B**: β-catenin expression is positively correlated with DKK1 level in 71 human HCC tissues. The median of all 71 cases was chosen as the cutoff point for separating low-DKK1 (n = 36) from high-DKK1 expressing tumors (n =35). **C**: DKK1 overexpression markedly enhanced the mRNA and protein level of β-catenin in HepG2 cells. GAPDH and β-actin served as internal controls. **D**: up-regulation of DKK1 expression enhanced the cytoplasmic/nuclear β-catenin accumulation in HepG2 tumor cells (original magnification, 100×). The expression level of β-catenin protein was detected with rabbit polyclonal antibody and revealed by Cy3-conjugated secondary antibody (red). The nuclei were counterstained with Hoechst33342 (blue). **E**: Restoration of DKK1 promoted the β-catenin-dependent transcriptional activity. Tumor cells were transfected with firefly luciferase reporter plasmids containing either wild-type or mutant TCF binding sites (indicated as Wt or Mut on the X axis), and a Renilla luciferase expressing construct. The firefly luciferase activity of each sample was normalized to the Renilla luciferase activity. The normalized luciferase activity of Vector-transfectants in each experiment was set as relative luciferase activity. ** P < 0.01, compared with Vector-transfectants. **F, G**: Treatment with β-catenin inhibitor IWP-2 (1 μM) attenuated the pro-migration and pro-invasion effect of DKK1. In **(F)**, after scratching a wound and removing the floating cells, tumors cells without treatment (panel 1, 3) or incubated with 1 μM IWP-2 (panel 2, 4) were applied to the wound scratch assay for 24 h. In **(G)** tumors cells treated as in **(F)** were applied to transwell chamber coated with Matrigel and then incubated for 24 h. The data are represented as means ± SEM. *, p <0.05; **, p <0.01.

**Figure 5:**
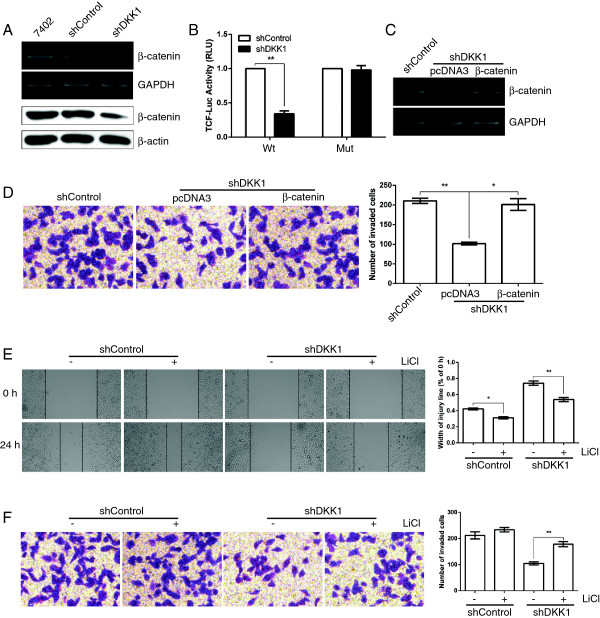
**Beta-catenin is involved in the dual promotive function of DKK1 on tumor migration and invasion. A**: Knockdown of DKK1 repressed the mRNA and protein level of β-catenin in Bel7402 cells. **B**: Silencing of DKK1 attenuated the β-catenin-dependent transcriptional activity. Tumor cells were transfected with firefly luciferase reporter plasmids containing either wild-type or mutant TCF binding sites (indicated as Wt or Mut on the X axis), and a Renilla luciferase expressing construct. The firefly luciferase activity of each sample was normalized to the Renilla luciferase activity. The normalized luciferase activity of shControl-transfectants in each experiment was set as relative luciferase activity 1. Therefore, no error bar is shown for shControl-transfectants. **, p < 0.01, compared with shControl-transfectants. **C**: Transient expression of β-catenin in Bel7402-shDKK1 cells was detected by RT-PCR analysis. Bel7402-shDKK1 cells transfected with pcDNA3 (lane 2) or pcDNA3-β-catenin (lane 3) were analyzed using RT-PCR. **D**: Restoration of β-catenin reversed the anti-invasion effect of shDKK1. **E, F**: Treatment with GSK3β inhibitor LiCl (5 mM) attenuated the anti-migration and anti-invasion effect of shDKK1. In **(E)**, after scratching a wound and removing the floating cells, tumors cells without treatment (panel 1, 3) or incubated with 5 mM LiCl (panel 2, 4) were applied to the wound scratch assay for 24 h. In **(F)** tumors cells treated as in **(E)** were applied to transwell chamber coated with Matrigel and then incubated for 24 h. The data are represented as means ± SEM. *, p <0.05; **, p <0.01.

Then we investigate a potential mechanism for DKK1-mediated HCC cell migration and invasion. It is well known that DKK1 is a typical inhibitor of Wnt/β-catenin signaling pathway, hence we examined that whether DKK1 was able to inhibit the expression of β-catenin. Firstly, we analyzed β-catenin expression using Immunohistochemical staining in 71 cases of human HCC tissues. Of great interest, the DKK1 level was positively correlated with β-catenin expression (Figure 4A and B). Consistently, introduction of DKK1 did not inhibit β-catenin expression, but remarkably increased the mRNA and protein level of β-catenin in HepG2 cells, compared with those of control cells (Figure 4C). In addition, by using immunofluorescence staining, we also observed that up-regulation of DKK1 expression noticeably promoted the cytoplasmic/nuclear β-catenin accumulation in HepG2 cells (Figure 4D). Furthermore, dual-luciferase reporter analysis also showed that restoration of DKK1 significantly promoted the activity of firefly luciferase that carried wildtype but not mutant TCF binding sites (Figure 4E). In contrast, the blockage of DKK1 dramatically attenuated the mRNA and protein level of β-catenin in Bel7402 cells (Figure 5A). Moreover, the activity of firefly luciferase that carried wildtype but not mutant TCF binding sites was dramatically attenuated when DKK1 was silenced in Bel7402 cells (Figure 5B). These findings indicated that β-catenin may be an important target downstream of DKK1.

### DKK1 exert its function by promoting β-catenin signaling

The role of β-catenin in DKK1-mediated phenotypes was then evaluated. First, we examined whether blockage of β-catenin could attenuated the effect of DKK1 expression. As expected, upon treatment with β-catenin inhibitor IWP-2, the promotive effect of DKK1 on migration and invasion was significantly attenuated in DKK1-transfectants, whereas there were no statistically significant differences in those of Vector-transfectants (Figure 4F and G). On the other hand, overexpression of β-catenin in shDKK1-transfectants restored the transcription of β-catenin (Figure 5C), and attenuated the inhibitory effect of shDKK1 on invasion (Figure 5D). As GSK3β is an important upstream target of β-catenin signaling, we further analyzed whether blockage of GSK3β could mimic the effect of β-catenin expression. Upon treatment with GSK3β inhibitor LiCl, the inhibitory effect of shDKK1 on migration (Figure 5E) and invasion (Figure 5F) was dramatically released.

**Figure 6:**
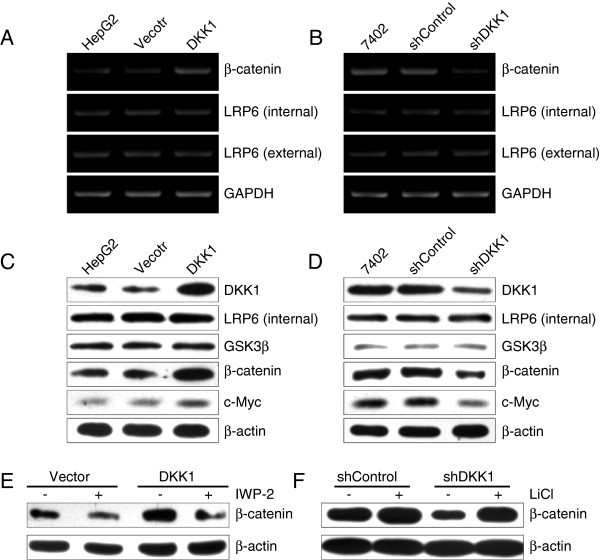
**DKK1 regulates β-catenin expression through the non-canonical Wnt pathway. A**: Restoration of DKK1 promoted the mRNA expression of β-catenin in HepG2 cells. **B**: Knockdown of DKK1 attenuated β-catenin mRNA expression in Bel7402 cells. **C**: Restoration of DKK1 promoted the protein levels of β-catenin and c-Myc in HepG2 cells. **D**: Knockdown of DKK1 attenuated the protein levels of β-catenin and c-Myc in Bel7402 cells. **E**: IWP-2 attenuated the positive effect of DKK1 on β-catenin expression in HepG2 cells. **F**: Treatment with GSK3β inhibitor LiCl restored the expression of β-catenin in Bel7402 cells.

It is well known that DKK1 antagonizes Wnt signaling by direct high-affinity binding to the extracellular domain of Wnt coreceptor lipoprotein receptor-related protein 6 (LRP6) and inhibiting the internalization of LRP6, which is responsible for the inhibition of GSK3β and activation of Wnt signaling cascade downstream. Therefore, two pairs of primers were selected to examine the mRNA expression of LRP6: one for the extracellular domain of LRP6, responsible for DKK1 binding, and another for the intracellular region, essential for inhibiting GSK3β [22]. Of great note, neither the extracellular domain nor the intracellular domain of LRP6 were changed, although the expression of β-catenin was dramatically altered upon DKK1 up-regulation (Figure 6A) or knockdown (Figure 6B). In addition, western blotting analysis also displayed that the protein level of LRP6 and GSK3β was not changed in comparison with those of control groups (Figure 6C and D). Whereas restoration of DKK1 significantly enhanced the expression of β-catenin, and the protein level of its downstream target c-Myc was also increased in HepG2 cells (Figure 6C). Vice versa, the blockage of DKK1 reduced the expression of β-catenin and c-Myc in Bel7402 cells (Figure 6D). Furthermore, upon β-catenin inhibitor IWP-2 treatment, the expression of β-catenin was attenuated (Figure 6E), which is consistent with the above observation on migration (Figure 4F) and invasion (Figure 4G), compared with the control. On the other hand, GSK3β inhibitor LiCl indeed recovered the expression of β-catenin in shDKK1-transfectants (Figure 6F), in agreement with the results of Figure 5E and F. These findings indicated that DKK1 may positively regulate the expression of β-catenin through a non-canonical Wnt signaling pathway.

### MMP7 is an important target downstream of DKK1/β-catenin signaling pathway

**Figure 7:**
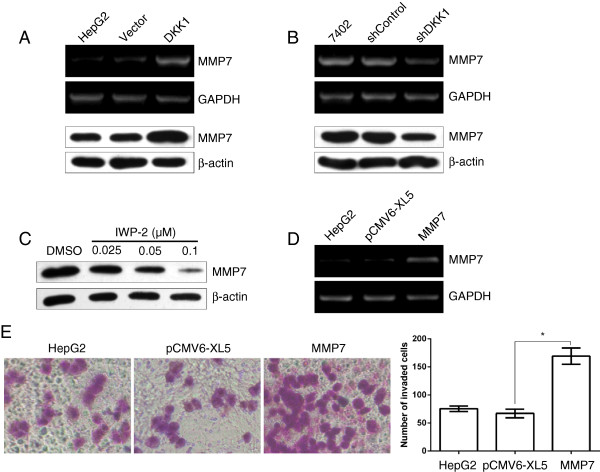
**DKK1 regulates the expression of MMP7. A**: Ectopic expression of DKK1 increased the mRNA and protein expression of MMP7 in HepG2 cells. HepG2 cells without transfection (lane 1) or transfected with Vector (lane 2) or DKK1 (lane 3) were analyzed using RT-PCR and western blotting. **B**: Knockdown of DKK1 suppressed the MMP7 mRNA and protein level in Bel7402 cells. RT-PCR and western blotting analysis were respectively used to detect the RNA and protein level of MMP7 in Bel7402 cells without transfection or cell transfected with shControl or shDKK1. GAPDH and β-Actin were used as internal controls. **C**: β-catenin inhibitor inhibited the expression of MMP7 in Bel7402 cells. Bel7402 cells were treated without or with various concentration of β-catenin inhibitor (IWP-2) for 24 h, and then the whole cell lysates were analyzed by western blotting for MMP7 expression, β-actin served as internal control. **D**: Transient expression of MMP7 in HepG2 cells detected by RT-PCR analysis. HepG2 cells without transfection (lane 1) or transfected with Vector (lane 2) or DKK1 (lane 3) were analyzed using RT-PCR. **E**: Upregulation of MMP7 mimicked the pro-invasion effect of DKK1 overexpression. HepG2 cells transfected with empty vector (pCMV6-XL5) or pCMV6-XL5-MMP7 were applied to transwell chamber coated with Matrigel. The data are represented as mean ± SEM. **, p <0.01.

Considering that MMP7 is one of the most important target genes downstream of β-catenin/TCF signaling, in addition, MMP7 has been shown to play a crucial role in promoting tumor cell migration and invasion [23]. We then investigate whether DKK1 regulated the expression of MMP7. As shown in Figure 7A and B, introduction of DKK1 significantly enhanced the mRNA and protein level of MMP7 in HepG2 cells (Figure 7A). Vice versa, when DKK1 was silenced by shRNA, the expression of MMP7 mRNA and protein were remarkably down-regulated in Bel7402 cells (Figure 7B). Moreover, upon treatment with β-catenin inhibitor IWP-2, the protein level of MMP7 was reduced in a dose-dependent manner (Figure 7C). Then we examined whether introduction of MMP7 could mimic the effect of β-catenin expression. As expected, HepG2 cells transiently transfected with MMP7 plasmids displayed a much invasive activity (Figure 7D and E), which phenocopied those of enhanced β-catenin expression. Taken together, our findings suggested that DKK1 promotes HCC cell migration and invasion at least partly by promoting β-catenin/MMP7 signaling.

## Discussion

In this work, we found that DKK1 is strongly overexpressed in both HCC cell lines and clinical HCC samples. The expression of DKK1 is positively associated with the migration and invasion, but did not influence the proliferation or colony formation of HCC. Mechanistically, we demonstrated that DKK1 promoted HCC cell migration and invasion by upregulating β-catenin/MMP7 pathway. During this manuscript in preparation, Tao *et al.* showed that increased DKK1 expression could promote HCC invasion and metastasis *via* up-regulation of RhoA and JNK expression and non-canonical Wnt pathway [15]. Their results in combination with our findings in this study, revealed the significant role of DKK1 in HCC cell migration and invasion.

Recently, Shen *et al.* demonstrated that DKK1 as a serum biomarker can improve the diagnostic accuracy for HCC in a large-scale, multicenter study. Their study revealed that the levels of DKK1 in serum are significantly higher in patients with HCC than in all controls [16]. In the present data, we found that DKK1 is expressed at high levels in 3 of 4 HCC cell lines in comparison with that of the control normal liver cell line. Furthermore, specific DKK1 was predominantly observed to be expressed in HCC tissues but not in the adjacent non-tumor liver tissues. All these findings indicated that DKK1 performs as an oncogenic factor rather than a tumor-suppressor in HCC.

Interestingly, in this study, we did not find that DKK1 expression influence HCC cell proliferation and colony formation, which are two of the most important features among the malignant cell behavior. While Qin *et al.* found that overexpression of DKK1 by transfection was able to inhibit the growth and migration in M-H7402 cells [14]. One possible explanation is that the impact of DKK1 on cell proliferation may vary in different cell types. Because the tumor cells they used is a metastatic subclone of human HCC H7402 cells by isolating from transplantation of H7402 cells into severe combined immunodeficient (SCID) mice [14]. In contrast, our experiments were performed by using two HCC cell lines, namely HepG2 and Bel7402.

Besides cell proliferation, migration and invasive capacity of cells are also two of the most important features of malignant cell behavior. Intriguingly, up-regulation of DKK1 significantly promoted HCC cell migration and invasion, vice versa, when DKK1 is repressed by a DKK1-specific shRNA, the tumor cell migration and invasion is markedly reduced. More importantly, in HCC tissues, the metastatic tumors showed much higher DKK1 expression comparison with that in the primary tumors. Consistent with our present data, Tung *et al.* found that knockdown of DKK1 significantly reduced both migratory and invasive abilities of the two independent SMMC7721 knockdown clones [17]. Along the same line of arguments, Tao *et al.* reported that DKK1 could promote HCC invasion and metastasis *in vitro* and *in vivo*[15]. Moreover, the increased expression of DKK1 closely correlates with multiple tumor nodes, high Edmondson-Steiner grade and vein invasion, as well as poor overall and disease-free survival of HCC [15]. Taken together, our results in combined with the findings from others, suggesting that DKK1 plays an important role in promoting HCC cell migration and invasion.

Deregulation of Wnt/β-catenin signaling pathway is a hallmark of major gastrointestinal cancers including HCC [24]. It is well-known that β-catenin is the chief effector downstream of this pathway. Approximately one third of HCCs have been reported to be associated with the aberrant expression of β-catenin [25]. β-catenin plays a pivotal role in regulating the HCC development and tumorigenesis through its association with E-cadherin in cell-cell adhesion, interaction with the hepatocyte growth factor (HGF) receptor c-Met and regulation the canonical Wnt/β-catenin signaling pathway [26]. DKK1 acts as a typical inhibitor of canonical Wnt/β-catenin pathway. Interestingly, several years ago, Yu *et al.* reported that the mRNA and protein expression levels of DKK1 are predominantly elevated in HCC. Moreover, the increased DKK1 expression is positively correlated with the total β-catenin accumulation in both HCC cell lines and clinical samples [27]. Along the same line of argument, Xu *et al.* recently reported that up-regulation of DKK1 in their study significantly correlates with the cytoplasmic/nuclear β-catenin accumulation in triple negative breast cancers [24]. Here, our data clearly suggested that DKK1 can directly promote tumor cell migration and invasion by stimulating β-catenin transcription and translation through a non-canonical Wnt signaling pathway in HCC cells. This conclusion is based on the following evidence. Firstly, DKK1 restoration not only markedly promotes both the level of β-catenin and β-catenin-dependent transcriptional activity, but also promotes the migration and invasion of HCC cells *in vitro*. Moreover, a positive relationship of DKK1 expression with β-catenin level was found in HCC tissues. Secondly, silencing of DKK1 not only significantly suppresses β-catenin expression and β-catenin-dependent transcriptional activity, but also attenuates the migration and invasion of HCC cells. Thirdly, neither the mRNA level of LRP6 nor the protein levels of LRP6 and GSK3β was affected by DKK1 expression.

MMP7, also known as matrilysin, is a secreted protein implicated in a broad range of extracellular matrix substrates destruction in various cancers. Additionally, MMP7 has been shown to play a crucial role for the invasive and metastatic potential of tumor cells [28,29]. Recently, several reports have shown that MMP7 is overexpressed in both the cells and tissues of HCC [30,31]. Moreover, it is well-known that MMP7 is one of the most important downstream target genes of β-catenin/TCF-4 [32]. In some tissues, β-catenin transcriptionally regulates MMP7. For example, β-catenin activation induces the expression and secretion of MMP7 and promotes the binding of T cell factor to the MMP7 promoter in kidney epithelial cells, whereas delivery of the gene encoding DKK1 abolishes its induction [32]. However, the relationship between MMP7 and DKK1 remain elusive in HCC. Here, we demonstrate MMP7 is an important target downstream of DKK1 in HCC cells, based on the following studies: overexpression of DKK1 or upon GSK3β inhibitor treatment is associated with enhanced level of MMP7, migration and invasion in HCC cells, whereas knockdown of DKK1 by shRNA represses MMP7 expression and inhibits migration and invasion of HCC cells. In addition, β-catenin inhibitor IWP-2 suppresses the protein level of MMP7 in a dose dependent manner and attenuates DKK1-mediated migration and invasion of HCC cells. All these findings suggested that DKK1 may enhance HCC cell migration and invasion by promoting β-catenin/MMP7 signaling pathway.

Taken together, our findings demonstrated that DKK1 promote HCC cell migration and invasion at least partly by promoting β-catenin/MMP7 signaling axis, which supports the notion that DKK1 could serve as a diagnostic biomarker for monitoring HCC development and progression. In addition, as DKK1 down-regulation can reduce the migration and invasion of HCC cells, it is a potential therapeutic strategy for advanced HCC.

## Competing interest

The authors declared that they have no competing interest.

## Authors’ contributions

SQX participated in the design of the study, data analysis, and manuscript writing. CJW participated in the design of the study and data analysis. LC participated in the design of the study, data analysis, and manuscript preparation. ML participated in the design of the study, data analysis, and manuscript preparation. QL participated in the design of the study, data analysis, and manuscript preparation. All authors read and approved the final manuscript.

## Supplementary Material

Additional file 1: Table S1 Primers for RT-PCR. Click here for file
