# Supplementary material for: DKK1 promotes hepatocellular carcinoma cell migration and invasion through β-catenin/MMP7 signaling pathway
Source: Mol Cancer. 2013 Dec 10;12:157. doi: 10.1186/1476-4598-12-157 (PMC4029244; doi:10.1186/1476-4598-12-157)
Supplement: Additional file 1: Table S1 — Primers for RT-PCR. [file 1476-4598-12-157-S1.doc]

**Supporting Table 1. Primers for RT-PCR**

| **Name** | **Sense Strand/Sense Primer (5'-3')** | **Antisense Strand/Antisense Primer (5'-3')** |
| --- | --- | --- |
| β-catenin | TACCATTCCATTGTTTGTGCAG | TGAAGAGAGAGCTGGTCAGCTC |
| LRP6(internal) | TTCAAACAGTCCTTCCAC | ATGAGGGCACAGGTTCTGAATCAT |
| LRP6(external) | AGACCATCAGCAGAGCCTTT | GCCCCACATTTGGAACTAAG |
| DKK1 | CTTTCTCCCTCTTGAGTCCTTCTG | CATAGCGTGACGCATGCAGCGTT |
| MMP7 | AGATGTGGAGTGCCAGATGT | TAGACTGCTACCATCCGTCC |
| GAPDH | CGGAGTCAACGGATTGGTCGTAT | AGCCTTCTCCATGGTGGTGAAGAC |

Data are expressed as the mean ± standard error of the mean (SEM) from at least three independent experiments.

**standard error of the mean**

(in statistics) an indication of how well the mean of a sample estimates the mean of a population. It is measured by the standard deviation of the means of randomly drawn samples of the same size as the sample in question.

**standard deviation**

A statistic used as a measure of the dispersion or variation in a distribution, equal to the square root of the arithmetic mean of the squares of the deviations from the arithmetic mean.
